# Associations between asthma and Life’s Essential 8: a cross-sectional study

**DOI:** 10.3389/fmed.2025.1446900

**Published:** 2025-02-10

**Authors:** Jiao Xu, Jianlei Tang

**Affiliations:** ^1^Department of Respiratory and Critical Care Medicine, WuJin Hospital Affiliated with Jiangsu University, WuJin Clinical College of Xuzhou Medical University, Changzhou, Jiangsu, China; ^2^Rehabilitation Department, WuJin Hospital Affiliated with Jiangsu University, WuJin Clinical College of Xuzhou Medical University, Changzhou, Jiangsu, China

**Keywords:** Life’s Essential 8, asthma, health behaviors score, health factors score, odd ratios

## Abstract

**Background:**

Asthma is a serious respiratory disease attributed to multiple factors. The Life’s Essential 8 (LE8), introduced by the American Heart Association, aims to improve and maintain cardiovascular health. However, the correlation between LE8 components and asthma remains unclear. We hypothesized that LE8 is a protective factor against asthma.

**Materials and methods:**

Multiple logistic regression analysis, restricted cubic spline (RCS) analysis, and subgroup analysis were used to analyze the data collected from the National Health and Nutrition Examination Survey (NHANES) from 2001 to 2018.

**Results:**

A total of 3,360 participants with asthma were included in the analysis. With all confounders controlled for, LE8 scores were negatively correlated with asthma prevalence (odds ratio (OR) per 10-point increment, 0.85 [95% confidence interval (CI), 0.82–0.88]). Compared to low LE8 scores, moderate and high LE8 scores were associated with reduced asthma risk, with adjusted ORs (95% CIs) of 0.59 (0.51–0.68) and 0.48 (0.39–0.58), respectively. Non-linear correlations were observed between LE8 scores and asthma (*p* non-linear = 0.01) and between health factor scores and asthma (*p* non-linear = 0.01). However, a linear dose–response correlation was noted between health behavior scores and asthma (*p* non-linear = 0.30). Subgroup analysis showed no significant interaction effects (*p* > 0.05), except in the sex and drinking status subgroups (*p* for interaction = 0.02).

**Conclusion:**

Asthma is associated with components of LE8, which warrants further attention and may contribute to reducing asthma prevalence.

## Introduction

As one of the most rapidly growing diseases, asthma affects 300 million patients worldwide and causes approximately 2.5 million fatalities annually (1, 2). An American study reported an increase of $3,266 in annual medical costs for one patient, and the estimated cost for treatment of asthma is more than $80 billion annually (3). Moreover, severe asthma can have a serious negative impact on an individual’s life, physical activity, and work. Hence, asthma takes a tremendous burden on individuals and societies, amplifying the challenges for prevention.

The roles of environmental factors, hereditary factors, comorbidity, and social behaviors are all related to the incidence, prevalence, and mortality of asthma (4). Mounting research has indicated there is an interconnected association between cardiovascular disease (CVD) and asthma (5). The risk of CVD in asthma patients is 42% higher than that in non-asthma individuals (6). A meta-analysis study revealed that the risk of the failure of heart function, myocardial infarction, atrial fibrillation, and diseases of coronary arteries increases in asthma patients (7, 8). There are multiple common risk factors for asthma and CVD, including obesity, smoking, environmental exposure, physical inactivity, and stress (7). Furthermore, chronic inflammation is the common pathogenesis for asthma and CVD (5, 9). Therefore, improving cardiovascular health (CVH) may be helpful in asthma prevention.

In 2022, the American Heart Association introduced Life’s Essential 8 (LE8) as a new indicator for monitoring cardiovascular health to replace Life’s Simple 7 (10). LE8 comprises four health behaviors, including diet, sleep health, physical activity, and nicotine exposure, and four health factors, including body mass index (BMI), blood lipids, blood glucose, and blood pressure (11). Although previous studies have investigated the association between asthma and some components of LE8, the overall effect on asthma is not clear. This cross-sectional study aims to explore our hypothesis that LE8 can reduce asthma incidence.

## Materials and methods

### Study design and participants

The study was conducted using data from the National Health and Nutrition Examination Survey (NHANES), which is a cross-sectional survey with stratification and a multi-stage probability sampling design (12). The study was performed with approval from the National Center for Health Statistics Research Ethics Review Board, and the written informed consents were signed by all participants (13). All the databases can be accessed from the NHANES website.^1^

**Figure 1:**
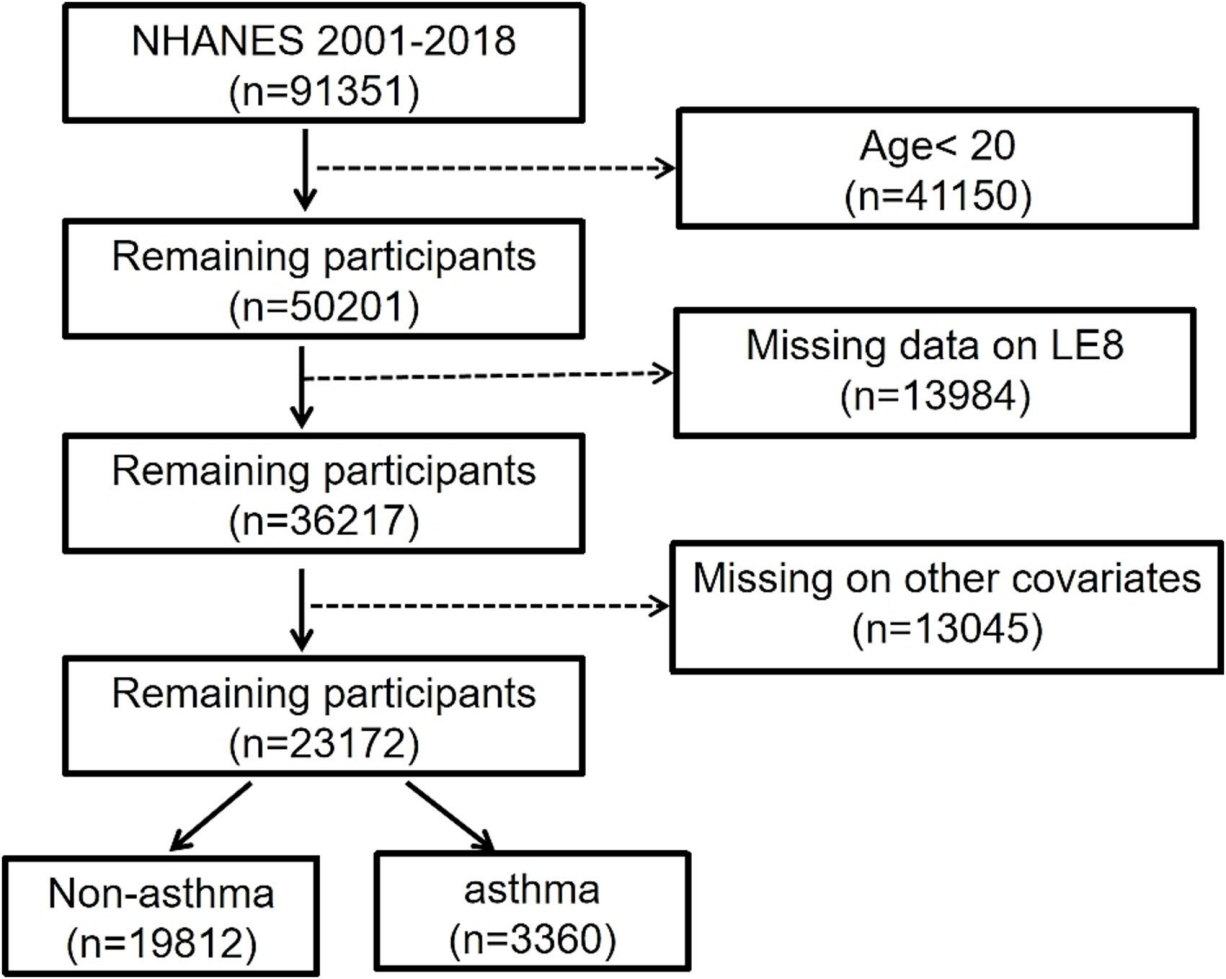
The flowchart of the study.

In total, 91,351 participants from the NHANES during 2001–2018 were first screened. These participants fitting the following criteria were then excluded: < 20 years of age (*n* = 41,150), no LE8 data (*n* = 13,984), and no other covariates (*n* = 13,045). Ultimately, 23,172 participants were enrolled, including 3,360 individuals with asthma confirmed by a certificated specialist and 19,812 individuals without asthma (Figure 1).

### Data and definitions in the NHANES

If the participant answered “Yes” to the question “Ever been told you have asthma?” in the questionnaire (MCQ010), the participant was identified as an asthma individual (14). The eight components of LE8 (11) were investigated according to a previous study (15). Dietary data were calculated using the Healthy Eating Index (HEI)-2015, with the first 24-h recall interview (16). The self-administered paper questionnaires collected the sleep duration, nicotine exposure, and physical activity. Non-high-density lipoprotein cholesterol (non-HDL) and blood glucose in fasting status were obtained from the laboratory, while blood pressure, height, and body weight were obtained from the mobile examination center. According to the presidential advisory published by the American Heart Association, each metric had an algorithm range of 0–100 points, allowing the generation of LE8 scores. As the unweighted mean of the eight components, LE8 scores were calculated from 0 to 100 points and assigned to different levels of CVH, i.e., 0–49 points too low CVH, 50–79 points to moderate CVH, and 80–100 points to high CVH (17). The detailed scoring methods of LE8 are shown in Table 1, and if all eight components of LE8 were shown as “NA,” the data was excluded. The covariates in the study, which were referred to in the previous asthmatic surveys, included sex (male/female), age (years), level of education, race, family income status, smoking status (never, former, now), drinking status (never, former, mild, moderate, heavy) and coronary heart disease status (yes or no) (18, 19). Age was grouped as 20–39, 40–59, and ≥ 60 years. Family income level was classified as low (PIR ≤ 1.3), moderate (1.3 < PIR < 3.5), and high (PIR ≥ 3.5) (20). Smoking status was classified as never smoker (< 100 cigarettes in a lifetime), former smoker (≥100 cigarettes in history but 0 currently), or current smoker (≥100 cigarettes in history and regular smoking currently) (21). There were five drinking statuses: never (<12 drinks total); former (≥12 drinks 1 year ago); mild (one drink for women and two drinks for men per day); moderate (≥2 drinks for females and ≥ 3 drinks for males per day, or 2 ≤ binge drinking <5 per month); heavy drinker (≥3 drinks for females and ≥ 4 drinks for males per day; or binge drinking ≥4 drinks for females and ≥ 5 drinks for males on the same occasion) (22, 23). BMI was classified as under/normal weight with BMI < 25, overweight with 25 ≤ BMI < 30, and obese with BMI ≥ 30 (24). Coronary heart diseases were defined when participants answered YES to “Ever told you had” of the respective conditions.

**Table 1:** Scoring of LE8.

Domain	CVH metric	Measurement	Quantification and scoring of CVH metric	
Health behaviors	Diet	Healthy Eating Index-2015 diet score percentile	Quantiles of DASH-style diet adherence *Scoring (Population):*	
			Points	Quantile
			100	≥95^th^ percentile (top/ideal diet)
			80	75^th^–94^th^ percentile
			50	50^th^–74^th^ percentile
			25	25^th^–49^th^ percentile
			0	1^st^–24^th^ percentile (bottom/least ideal quartile)
	Physical activity	Self-reported minutes of moderate or vigorous physical activity per week	*Metric*: Minutes of moderate (or greater) intensity activity per week	
			Scoring	
			Points	Minutes
			100	≥150
			90	120–149
			80	90–119
			60	60–89
			40	30–59
			20	1–29
			0	0
	Nicotine exposure	Self-reported use of cigarettes or inhaled nicotine-delivery systems	*Metric*: Combustible tobacco use and/or inhaled NDS use or secondhand smoke exposure	
			Scoring	
			Points	Status
			100	Never smoker
			75	Former smoker, quit ≥5 years
			50	Former smoker, quit 1–<5 years
			25	Former smoker, quit <1 year, or currently using inhaled NDS
			0	Current smoker
			Subtract 20 points (unless the score is 0) for living with an active indoor smoker in the home	
	Sleep health	Self-reported average hours of sleep per night	*Metric*: Average hours of sleep per night	
			Scoring	
			Points	Level
			100	7–<9
			90	9–<10
			70	6–<7
			40	5–<6 or ≥ 10
			20	4–<5
			0	< 4
Health factors	Body mass index	Body weight (kg) divided by height squared (m^2^)	*Metric*: Body mass index (kg/m^2^)	
			Scoring	
			Points	Level
			100	< 25
			70	25.0–29.9
			30	30.0–34.9
			15	35.0–39.9
			0	≥ 40.0
	Blood lipids	Plasma total and HDL-cholesterol with the calculation of non-HDL-cholesterol	*Metric*: Non-HDL-cholesterol (mg/dL)	
			Scoring	
			Points	Level
			100	< 130
			60	130–159
			40	160–189
			20	190–219
			0	≥ 220
			If the drug-treated level, subtract 20 points	
	Blood glucose	Fasting blood glucose or casual hemoglobin A1c	*Metric*: Fasting blood glucose (mg/dL) or Hemoglobin A1c (%)	
			Scoring	
			Points	Level
			100	No history of diabetes and FBG <100 (or HbA1c < 5.7)
			60	No diabetes and FBG 100–125 (or HbA1c 5.7–6.4) (Pre-diabetes)
			40	Diabetes with HbA1c <7.0
			30	Diabetes with HbA1c 7.0–7.9
			20	Diabetes with HbA1c 8.0–8.9
			10	Diabetes with Hb A1c 9.0–9.9
			0	Diabetes with HbA1c ≥10.0
	Blood pressure	Appropriately measured systolic and diastolic blood pressure	*Metric*: Systolic and diastolic blood pressure (mm Hg)	
			Scoring	
			Points	Level
			100	< 120/<80 (Optimal)
			75	120–129/<80 (Elevated)
			50	130–139 or 80–89 (Stage I HTN)
			25	140–159 or 90–99
			0	≥ 160 or ≥ 100
			Subtract 20 points if treated level	

### Statistical methods

Considering NHANES sampling weights, the data were analyzed by R (version 4.3.2). First, the baselines of the participants were evaluated by univariate analysis. The categorical data were expressed as percentages (%) for statistical analysis with a weighted Chi-square test, while continuous variables were analyzed by weighted t-test and shown as the mean ± errors (SE). Multivariate logistic regression analysis was performed to analyze the associations between LE8 groups and asthma, represented by odds ratios (ORs) and 95% confidence intervals (CIs). There were three statistical models: the univariate model (no adjustment of covariates), Model I (with adjustment of education level, age, sex, race, and family income status), and Model II (with adjustment of all covariates). Next, whether non-linear relationships existed between LE8 and asthma was investigated by restricted cubic spline (RCS) analyses. 4 knots were selected to balance the smoothness of the curve and avoid the loss of accuracy caused by overfitting. Finally, subgroup analysis was performed to determine the interaction between the stratified factors and LE8 scores. A two-tailed *p-*value of <0.05 was considered the statistical significance level.

## Results

### Characteristics of participants

There were 23,172 participants from the NHANES during 2001–2018 who were finally enrolled in this study, including 3,360 asthma patients. The age, sex, education level, race, poverty income ratio, smoking status, drinking status, BMI, LE8 score, HEI-2015 diet, nicotine exposure condition, sleep health, body mass index, and blood glucose level in the asthma group were significantly different from the non-asthma group (*p* < 0.05), while coronary heart disease, physical activity level, blood lipids score and blood pressure level were not statistically different between the two groups (*p >* 0.05) (Table 2).

**Table 2:** Baseline characteristics of patients with asthma in the NHANES (2001–2018).

Characteristics	Total	Non-asthma	Asthma	*p* value
Age (SE)	47.69 (0.26)	48.04 (0.27)	45.69 (0.41)	< 0.0001
Age Group *n* (%)^a^				< 0.0001
20–39	7,612 (34.84)	6,346 (33.93)	1,266 (40.17)	
40–59	7,833 (39.08)	6,716 (39.55)	1,117 (36.35)	
≥60	7,727 (26.08)	6,750 (26.53)	977 (23.48)	
Sex *n* (%)^a^				< 0.0001
Female	11,769 (51.41)	9,838 (50.17)	1931 (58.62)	
Male	11,403 (48.59)	9,974 (49.83)	1,429 (41.38)	
Education level *n* (%)^a^				0.03
Lower than high school	1933 (4.13)	1715 (4.26)	218 (3.38)	
High school	8,370 (32.43)	7,196 (32.68)	1,174 (30.96)	
Higher than high school	63.44 (0.02)	10,901 (63.06)	1968 (65.66)	
Race *n* (%)^a^				< 0.0001
Non-Hispanic White	10,889 (71.57)	9,211 (71.41)	1,678 (72.45)	
Non-Hispanic Black	4,661 (9.77)	3,882 (9.49)	779 (11.44)	
Mexican American	3,382 (7.44)	3,073 (7.90)	309 (4.77)	
Other Race	4,240 (11.22)	3,646 (11.20)	594 (11.34)	
Family gross income *n* (%)^a^				< 0.0001
Low	6,792 (19.11)	5,605 (18.21)	1,187 (24.40)	
middle	8,763 (35.45)	7,609 (35.83)	1,154 (33.24)	
High	7,617 (45.44)	6,598 (45.97)	1,019 (42.36)	
Smoking status *n* (%)^a^				< 0.0001
Never	12,723 (55.20)	11,029 (55.80)	1,694 (51.75)	
Former	5,843 (25.69)	4,990 (25.75)	853 (25.35)	
Now	4,606 (19.11)	3,793 (18.46)	813 (22.90)	
Drinking status *n* (%)^a^				0.04
Never	3,009 (10.08)	2,643 (10.35)	366 (8.52)	
Former	3,823 (13.45)	3,244 (13.30)	579 (14.36)	
Mild	8,069 (37.86)	6,922 (38.05)	1,147 (36.79)	
Moderate	3,704 (18.04)	3,131 (17.99)	573 (18.33)	
Heavy	4,567 (20.57)	3,872 (20.33)	695 (21.99)	
BMI (SE)	29.00 (0.09)	28.79 (0.09)	30.23 (0.21)	< 0.0001
BMI Group *n* (%)^a^				< 0.0001
Normal	6,601 (29.60)	5,769 (30.06)	832 (26.88)	
Obese	8,863 (37.22)	7,283 (36.05)	1,580 (44.03)	
Overweight	7,708 (33.19)	6,760 (33.89)	948 (29.09)	
PIR (SE)	3.11 (0.04)	3.14 (0.03)	2.94 (0.05)	< 0.0001
Coronary heart disease *n* (%)^a^				0.1
No	22,205 (96.53)	19,004 (96.66)	3,201 (95.80)	
Yes	967 (3.47)	808 (3.34)	159 (4.20)	
LE8 score (SE)	68.45 (0.24)	68.82 (0.24)	66.32 (0.44)	< 0.0001
HEI-2015 diet score (SE)	39.45 (0.46)	39.94 (0.47)	36.58 (0.95)	< 0.001
Physical activity score (SE)	72.15 (0.48)	72.32 (0.52)	71.12 (0.97)	0.26
Nicotine exposure score (SE)	71.55 (0.50)	72.19 (0.48)	67.84 (1.12)	< 0.0001
Sleep health level (SE)	83.61 (0.28)	84.41 (0.26)	78.96 (0.64)	< 0.0001
Body mass index (SE)	60.57 (0.42)	61.45 (0.44)	55.41 (0.98)	< 0.0001
Blood lipids level (SE)	64.28 (0.34)	64.10 (0.36)	65.38 (0.71)	0.08
Blood glucose level (SE)	86.31 (0.24)	86.51 (0.26)	85.17 (0.57)	0.03
Blood pressure level (SE)	69.71 (0.35)	69.65 (0.38)	70.12 (0.67)	0.51

The data were expressed as the mean ± SD for continuous variables or percentages for categorical variables. *p* values were calculated by weighted t-tests and weighted chi-square tests. ^a^Un-weighted frequency counts and weighted percentages were shown.

### LE8 score and asthma

The univariate analysis model indicated that asthma is significantly correlated with the LE8 score, with ORs of 0.59 (95% CI 0.51, 0.69) for the moderate LE8 group and 0.55 (95% CI 0.46, 0.66) for the high LE8 group compared to the low LE8 group. Additionally, for each 10-point increase in the L38 score, the OR was 0.89 (95% CI 0.85, 0.92).

**Figure 2:**
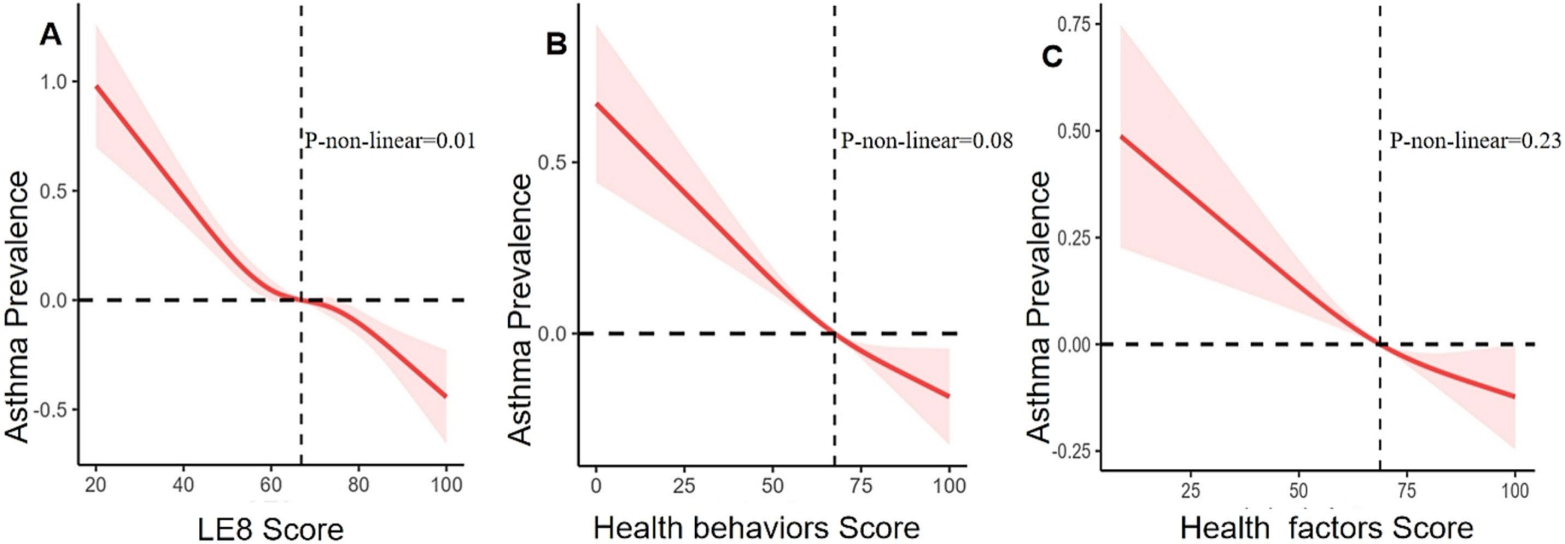
Relationships between asthma prevalence and LE8 score **(A)**, health behaviors score **(B)**, and health factors score **(C)**. ORs (solid lines) and 95% CIs (shaded areas) were adjusted for sex, age, education level, race, family income status, drinking status, and coronary heart disease.

After adjustment using multivariate analysis model 1, the ORs for asthma were 0.57 (95% CI 0.49, 0.66) in the moderate LE8 group and 0.46 (95% CI 0.38, 0.56) in the high LE8 group compared to the low LE8 group, while the OR for each 10-point increase in the LE8 score was 0.84 (95% CI 0.81, 0.87). When all covariates were adjusted using multivariate analysis model 2, the ORs were 0.59 (95% CI 0.51, 0.68) for the moderate LE8 group and 0.48 (95% CI 0.39, 0.58) for the high LE8 group, with an OR of 0.85 (95% CI 0.82, 0.88) for each 10-point increase in the LE8 score, compared to the low LE8 group (Table 3). A non-linear association was observed between the LE8 score and asthma (p for non-linearity = 0.01, Figure 2).

**Table 3:** Association of the LE8 scores with asthma.

	Univariate model		Model 1		Model 2	
	OR (95% CI)	*p* value	OR (95% CI)	*P* value	OR (95% CI)	*P* value
LE8 scores						
Low (0–49)	1.00 (Reference)		1.00 (Reference)		1.00 (Reference)	
Moderate (50–79)	0.59 (0.51,0.69)	<0.0001	0.57 (0.49,0.66)	<0.0001	0.59 (0.51,0.68)	<0.0001
High (80–100)	0.55 (0.46,0.66)	<0.0001	0.46 (0.38,0.56)	<0.0001	0.48 (0.39,0.58)	<0.0001
Per 10-point increase	0.89 (0.85,0.92)	<0.0001	0.84 (0.81,0.87)	<0.0001	0.85 (0.82,0.88)	<0.0001
Health behaviors score						
Low (0–49)	1.00 (Reference)		1.00 (Reference)		1.00 (Reference)	
Moderate (50–79)	0.70 (0.62,0.80)	<0.0001	0.71 (0.62,0.81)	<0.0001	0.73 (0.64,0.83)	<0.0001
High (80–100)	0.60 (0.51,0.71)	<0.0001	0.61 (0.51,0.72)	<0.0001	0.62 (0.53,0.73)	<0.0001
Per 10-point increase	0.91 (0.88,0.93)	<0.0001	0.91 (0.89,0.94)	<0.0001	0.91 (0.89,0.94)	<0.0001
Health factors score						
Low (0–49)	1.00 (Reference)		1.00 (Reference)		1.00 (Reference)	
Moderate (50–79)	0.78 (0.67,0.91)	0.002	0.74 (0.63,0.86)	<0.001	0.75 (0.64,0.87)	<0.001
High (80–100)	0.81 (0.70,0.94)	0.01	0.63 (0.54,0.75)	<0.0001	0.64 (0.54,0.76)	<0.0001
Per 10-point increase	0.91 (0.88,0.93)	<0.0001	0.91 (0.89,0.94)	<0.0001	0.91 (0.89,0.94)	<0.0001

Model 1 was adjusted for age, sex, education level, race, and family income status. Model 2 was adjusted for age, sex, education level, race, family income status, drinking status, and coronary heart disease.

### Health behavior scores and asthma

The univariate analysis model indicated that asthma is significantly correlated with the health behavior score, with ORs of 0.70 (95% CI 0.62, 0.80) in the moderate health behavior group and 0.60 (95% CI 0.51, 0.71) in the high health behavior group, compared to the low health behavior group. Additionally, for each 10-point increase in the health behavior score, the OR was 0.91 (95% CI 0.88, 0.93).

After adjustment using multivariate analysis model 1, the ORs for asthma were 0.71 (95% CI 0.62, 0.81) in the moderate health behavior group and 0.61 (95% CI 0.51, 0.72) in the high health behavior group, compared to the low health behavior group. For each 10-point increase in the health behavior score, the OR was 0.91 (95% CI 0.89, 0.94).

In multivariate analysis model 2, the ORs for asthma were 0.73 (95% CI 0.64, 0.83) in the moderate behavior group and 0.62 (95% CI 0.53, 0.73) in the high health behavior group, compared to the low health behavior group. The OR for each 10-point increase in the health behavior score was 0.91 (95% CI 0.89, 0.94) (Table 3). However, health behavior scores and asthma did not show a non-linear association (*p* = 0.08, Figure 2).

### Health factors scores and asthma

The univariate analysis model indicated that asthma is significantly correlated with the health factor score, with ORs of 0.78 (95% CI 0.67, 0.91) in the moderate health factor group and 0.81 (95% CI 0.70, 0.94) in the high health factor group, compared to the low health factor group. Additionally, for each 10-point increase in the health factor score, the OR was 0.91 (95% CI 0.88, 0.93).

After adjustment using multivariate analysis model 1, the ORs for asthma were 0.74 (95% CI 0.63, 0.86) in the group with moderate health factor and 0.63 (95% CI 0.54, 0.75) in the group with high health factor, compared to the low health factor group. For each 10-point increase in the health score, the OR was 0.91 (95% CI 0.89, 0.94).

After full adjustment with multivariate analysis model 2, the ORs for asthma were 0.75 (95% CI 0.64, 0.87) in the group with moderate health factors and 0.64 (95% CI 0.54, 0.76) in the group with high health factors, while the OR for each 10-point increase in health factor scores was 0.91 (95% CI 0.89, 0.94), compared to the group with low health factors (Table 3). There was no linear association between health factor scores and asthma (non-linear *p* = 0.23, Figure 2).

### Components of LE8 and asthma

In a fully adjusted model, asthma showed significantly negative associations with sleep health levels and blood glucose levels (Table 4). The risk of asthma was significantly lower in the group with moderate sleep health level (6–7 average hours of sleep per night) and the group with high sleep health level (7–10 average hours of sleep per night) when compared with the group with low sleep health level (<6 or ≥ 10 average hours of sleep per night). The OR for asthma in a per-10-point increase of sleep health level was 0.92 (95% CI, 0.91–0.94). Additionally, the risk of asthma was significantly lower in the group with moderate blood glucose levels (no diabetes, fasting blood glucose 100–125 [or glycosylated hemoglobin 5.7–6.4]) and the group with high blood glucose levels (no history of diabetes, fasting blood glucose <100 [or glycosylated hemoglobin <5.7]), compared with the group with low blood glucose levels (people with diabetes). The OR for asthma in a 10-point increase of blood glucose level was 0.95 (95% CI, 0.93–0.97).

**Table 4:** Associations between components of LE8 and asthma.

Character	Cases/participants	OR (95% CI)	*P* value
HEI-2015 diet score			
Low (0–49)	1811/11607	1.00 (Reference)	
Moderate (50–79)	788/5797	0.87 (0.77,0.98)	0.03
High (80–100)	761/5768	0.87 (0.75,1.00)	0.05
Per 10-point increase	3360/23172	0.97 (0.95,0.99)	0.005
Physical activity score			
Low (0–49)	1053/7177	1.00 (Reference)	
Moderate (50–79)	168/1093	0.97 (0.76,1.23)	0.79
High (80–100)	2139/14902	0.94 (0.84,1.05)	0.28
Per 10-point increase	3360/23172	0.99 (0.98,1.00)	0.16
Nicotine exposure score			
Low (0–49)	913/5239	1.00 (Reference)	
Moderate (50–79)	753/5210	0.95 (0.80,1.12)	0.53
High (80–100)	1694/12723	0.81 (0.71,0.92)	0.002
Per 10-point increase	3360/23172	0.98 (0.97,0.99)	0.003
Sleep health score			
Low (0–49)	802/4011	1.00 (Reference)	
Moderate (50–79)	772/4969	0.71 (0.62,0.81)	<0.0001
High (80–100)	1786/14192	0.58 (0.52,0.65)	<0.0001
Per 10-point increase	3360/23172	0.92 (0.91,0.94)	<0.0001
BMI score			
Low (0–49)	1580/8863	1.00 (Reference)	
Moderate (50–79)	948/7708	0.74 (0.65,0.84)	<0.0001
High (80–100)	832/6601	0.68 (0.59,0.78)	<0.0001
Per 10-point increase	3360/23172	0.95 (0.93,0.96)	<0.0001
Blood lipids score			
Low (0–49)	1097/7809	1.00 (Reference)	
Moderate (50–79)	717/5355	0.93 (0.81,1.07)	0.29
High (80–100)	1546/10008	1.00 (0.90,1.11)	0.99
Per 10-point increase	3360/23172	1.00 (0.98,1.01)	0.61
Blood glucose score			
Low (0–49)	548/3014	1.00 (Reference)	
Moderate (50–79)	646/4870	0.71 (0.59,0.85)	<0.001
High (80–100)	2166/15288	0.64 (0.53,0.76)	<0.0001
Per 10-point increase	3360/23172	0.95 (0.93,0.97)	<0.0001
Blood pressure score			
Low (0–49)	789/5335	1.00 (Reference)	
Moderate (50–79)	1025/7561	0.86 (0.75,0.98)	0.02
High (80–100)	1546/10276	0.82 (0.70,0.95)	0.01
Per 10-point increase	3360/23172	0.97 (0.96,0.99)	0.004

HEI, Healthy Eating Index; LE8, Life’s Essential 8; OR, odds ratio. Adjusted for sex, age, education level, race, family income status, drinking status, and coronary heart disease.

### Subgroup analysis

**Figure 3:**
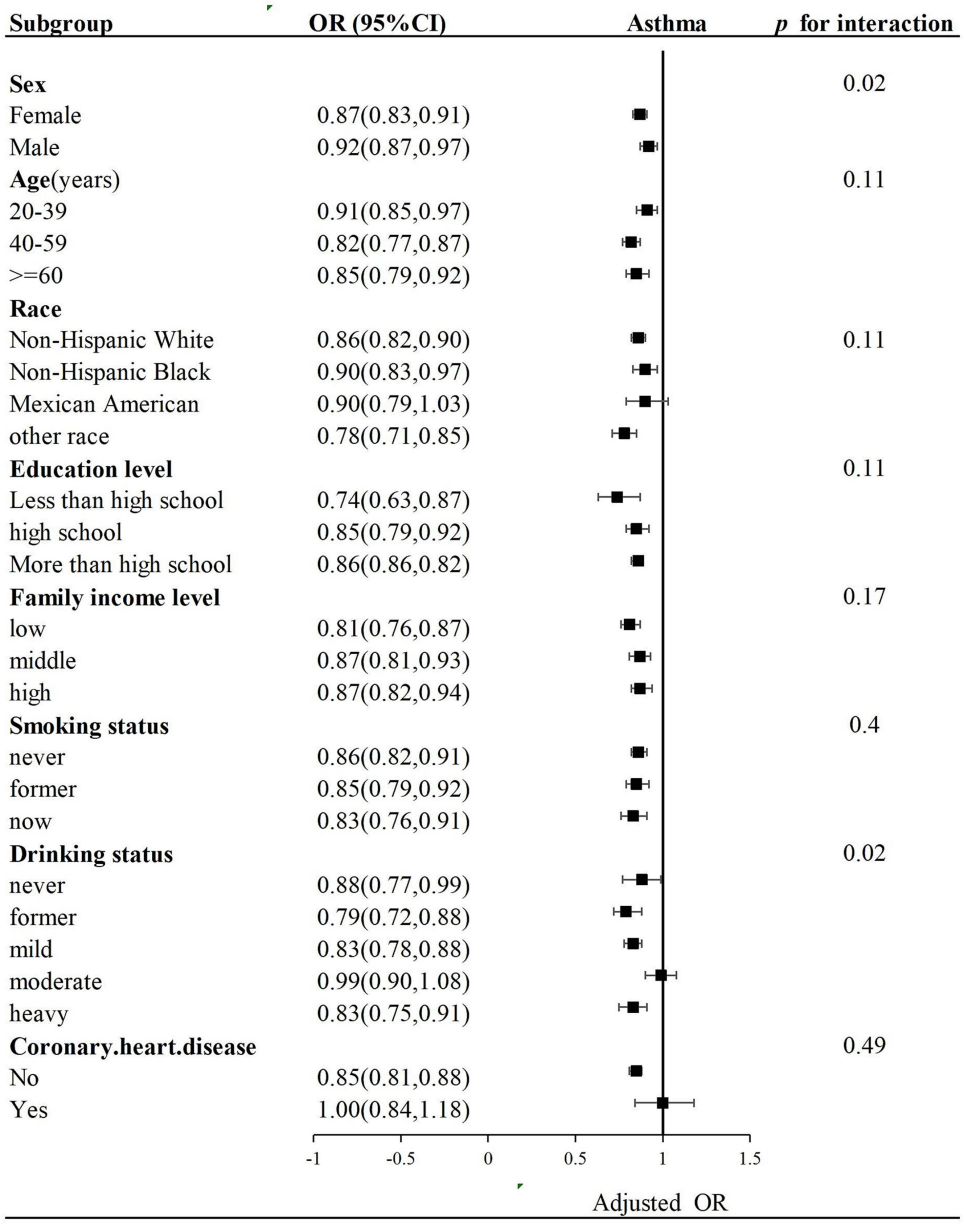
Subgroup analysis of the association of LE8 scores and asthma. OR was calculated as per a 10-point increase in LE8 score. Each stratification was adjusted for sex, age, education level, race, family income status, drinking status, and coronary heart disease.

Subgroup analysis with stratification of sex, age, education level, race, family gross income, drinking status, and coronary heart disease was performed. Complications such as hypertension, diabetes, and obesity (BMI ≥ 30 kg/m^2^), which were shown in Table 1, have been included in LE8 components and assumed as parts of variables. Hence, we did not explore their interaction effects on LE8 components. The negative association between LE8 scores and asthma remained consistent in different categories, including age group, race, education level, family gross income, and coronary heart disease, but without significant interaction (*p* for interaction >0.05). However, LE8 scores and sex and drinking status with asthma demonstrated significant interactions (*p* for interaction = 0.02) (Figure 3).

## Discussion

Asthma is a major non-communicable respiratory disease that affected 262 million people and killed 455,000 patients in 2019 (25). This disease imposes a tremendous burden on individuals, public health systems, society, and the economy. Therefore, early prevention and effective management of asthma are critical and important tasks. The results of the present study suggest that LE8 is a comprehensive and easy-to-use tool to evaluate the risk of asthma, demonstrating that higher LE8 scores are associated with a lower incidence of asthma. Our study also proposes that maintaining optimal CVH could help reduce the onset of asthma and should be promoted as a strategy for the timely identification and management of asthma. In addition, LE8 scores could facilitate interdisciplinary cooperation, enabling a comprehensive assessment and optimal interventions for patients.

There has been an increase in the incidence of asthma over the past few decades, especially in developed Western countries (26). More and more studies have demonstrated that many lifestyles and factors may be related to asthma, and changing these factors could reduce asthma attacks. A high-fiber diet could decrease asthma prevalence, especially for women and specific racial groups (27). The offspring of women taking pregnant vitamin D have 40–50% less wheezing than babies without vitamin D during pregnancy (28, 29). In the Chinese population, short night sleep duration is reported to increase the number of asthma incidents (30). Very short sleep (<6 h) is found to be associated with more persistent asthma (31). Approximately 50% of adults having asthma are current or former smokers, and they have a 25-fold asthma incidence compared to non-smokers (32).

Furthermore, smoking is found to be associated with worse clinical outcomes of asthma (33). Physical activity is proposed to improve asthma control and health-related life quality and reduce incident asthma (34, 35). Obesity and asthma often coexist in the same person. Individuals who are obese have a 50% incidence of asthma (36). In addition, obesity can increase the prevalence of asthma and/or make asthma control difficult. Some comorbidities of obesity, i.e., type 2 diabetes, dyslipidemia, breathing disorder related to sleeping, or hypertension, can deteriorate asthma (37). A positive association is proved between asthma and type 1 diabetes (OR, 1.15; 95%CI, 1.06–1.25), and children with asthma tend to have higher triglyceride levels and higher insulin resistance (38, 39). Abnormal lipid metabolism is present in asthma, which is correlated with the severity and IgE levels (40). These studies indicated that asthma is a kind of disease attributed to multiple factors, including diet, sleep duration, smoking, physical activity, obesity, diabetes, dyslipidemia, et al., which can contribute to the susceptibility of asthma. However, the combined effect of all the factors in LE8 on asthma has not been comprehensively investigated.

The former CVH assessment tool Life’s Simple 7 has been applied in assessing non-communicable chronic diseases (NCDs). Based on health factors and health behaviors, the new CVH indicator LE8 reflects self-rated health and health-related quality of life. A high LE8 score shows a beneficial impact on chronic disorders, including nonalcoholic fatty liver disease, atherosclerotic CVD, chronic kidney disease, stroke, and dementia (41–46). In the UK Biobank, chronic obstructive pulmonary disease accounts for 40.0% of the top 10 NCDs in low-moderate CVH (<80 points) (47). A prospective cohort study suggested that low asthma-PRS (polygenic risk score) and high CVH have a minimal risk of adult-onset asthma (0.28, 95% CI: 0.23, 0.34) (48). Using NHANES data from the U.S., we investigated the dose–response association between LE8 and asthma, and the results indicated that higher LE8 scores, health behavior scores, and health factor scores demonstrated a significant association with a lower risk of asthma.

Meanwhile, higher sleep health scores and blood glucose levels demonstrated a significant association with a lower risk of asthma. These results indicated that addressing sleep difficulties and controlling glucose may benefit asthma patients. Blood lipids include triglycerides, phospholipids, glycolipids, sterols, and steroids, essential substances for living cells’ basic metabolism. While blood lipids in LE8 only include non-high-density lipoprotein cholesterol (HDL-C), this contradicts previous research. Compared to healthy patients, asthma patients spend less time doing moderate-to-vigorous physical activity and do very few daily steps (49). Participating in physical activity will increase the fear of provoking asthma symptoms, and those with serious asthma are prone to avoiding exercise (50, 51). In our study, only 13% (437) of 3,360 asthma patients had moderate and high physical activity scores, demonstrating that asthma patients lack enough physical activity. Besides, there are only a few limited forms of physical activity and greater heterogeneity in NHANES, such as daily, leisure time, and sedentary activities. At last, the physical activity score came from the physical activity questionnaire present with recall bias. There are sex-related differences in asthma (52). The prevalence of female asthma is 20% higher compared with male asthma. Moreover, asthma symptom attacks are more frequent and more common in women than in men (53–55).

In our previous study, a higher OBS had a stronger antioxidant exposure. OBS was negatively associated with female asthma, which meant reducing pro-oxidant factors simultaneously, such as smoking, drinking, and obesity, could reduce the prevalence of female asthma (18). Improving the LE8 score would likely significantly reduce the incidence of female asthma. A nonfunctional ALDH2 was found in 50% of Asians. Approximately half of Japanese asthma patients had asthma exacerbation after drinking alcohol, which was referred to as alcohol-induced asthma resulting from the increase of histamine plasma concentrations. Shimoda et al. investigated 55% of asthma patients, reduced 20% of forced expiratory volume in 1 s, and increased blood acetaldehyde and plasma histamine levels (55). However, in non-Asians, alcohol has also been proven to have a therapeutic effect on asthma. Alcohol may reduce the sensitivity of the airways, and chronic alcohol consumption may inhibit asthmatic immunity over time (56). Hence, the relationship between LE8 and asthma will be affected by alcohol for its effect on asthma attacks and alleviation of asthmatic symptoms. Overall, LE8 scores and asthma demonstrated stronger negative associations in middle-aged, other races, and no coronary heart disease populations.

The present study used a cross-sectional method to investigate the association between LE8 and asthma with representative data of American adults from the NHANES. We believe that LE8 is a risk for asthma onset, and improving LE8 scores would be good for asthma. However, some limitations in the present study should be pointed out. First, causal relationships cannot be identified in this cross-sectional study. The second limitation is that the definitions of most LE8 components are based on questionnaires, which would have a certain extent of recall bias. Another limitation was that the results were from the American asthma population, and there may be population and regional differences.

## Conclusion

LE8 scores and health factors demonstrated a negative, non-linear correlation with the prevalence of asthma, whereas health behavior scores were negatively and linearly associated with asthma. Notably, the investigation highlighted the importance of optimal sleep duration and normal glucose levels. These findings suggest that LE8, as a practical and comprehensive health indicator, may be useful for the early identification of asthma risk and for minimizing the burden of asthma. However, the causal relationship between LE8 and asthma requires further investigation, and the precise mechanisms linking LE8 to asthma remain to be clarified.

### Future directions

Overall, LE8 may be a simple and comprehensive tool for preventing asthma. The eight components of LE8, including diet, sleep health, physical activity, nicotine exposure, BMI, blood lipids, blood glucose, and blood pressure, can be obtained from clinical practice and simple questionnaires. More large-scale prospective clinical programs and randomized controlled studies can be conducted to comprehensively address the interactions between LE8 and asthma.

## Data Availability

The datasets presented in this study can be found in online repositories. The names of the repository/repositories and accession number(s) can be found at: https://www.cdc.gov/nchs/nhanes/?CDC_AAref_Val=https://www.cdc.gov/nchs/nhanes/index.htm.

## Footnotes

^1^ https://www.cdc.gov/nchs/nhanes/?CDC_AAref_Val=https://www.cdc.gov/nchs/nhanes/index.htm
